# *Plasmodium falciparum* K76T *pfcrt* Gene Mutations and Parasite Population Structure, Haiti, 2006–2009

**DOI:** 10.3201/eid2205.150359

**Published:** 2016-05

**Authors:** Macarthur Charles, Sanchita Das, Rachel Daniels, Laura Kirkman, Glavdia G. Delva, Rodney Destine, Ananias Escalante, Leopoldo Villegas, Noah M. Daniels, Kristi Shigyo, Sarah K. Volkman, Jean W. Pape, Linnie M. Golightly

**Affiliations:** Weill Medical College of Cornell University, New York, New York, USA (M. Charles, S. Das, L. Kirkman, K. Shigyo, J.W. Pape, L.M. Golightly);; The Haitian Group for the Study of Kaposi's Sarcoma and Opportunistic Infections Centers (GHESKIO);; Port-au-Prince, Haiti (M. Charles, G.G. Delva, R. Destine, J.W. Pape);; Harvard T.H. Chan School of Public Health, Boston, Massachusetts, USA (R. Daniels, S.K. Volkman);; Broad Institute, Cambridge, Massachusetts, USA (R. Daniels, S.K. Volkman);; Temple University, Department of Biology, Philadelphia, Pennsylvania, USA (A. Escalante);; ICF International, Rockville, Maryland, USA (L. Villegas);; Centro de Investigación de Campo Dr. Francesco Vitanza, Tumeremo, Venezuela (L. Villegas);; Massachusetts Institute of Technology, Cambridge (N.M. Daniels);; Simmons College, Boston (S.K. Volkman)

**Keywords:** Plasmodium falciparum, malaria, parasites, vector-borne infections, vectorborne, K76T pfcrt, chloroquine, CQR, CQS, gene mutation, haplotype, Hispaniola, Jeremie, Les Cayes, Haiti

## Abstract

Hispaniola is the only Caribbean island to which *Plasmodium falciparum* malaria remains endemic. Resistance to the antimalarial drug chloroquine has rarely been reported in Haiti, which is located on Hispaniola, but the K76T *pfcrt* (*P. falciparum* chloroquine resistance transporter) gene mutation that confers chloroquine resistance has been detected intermittently. We analyzed 901 patient samples collected during 2006–2009 and found 2 samples showed possible mixed parasite infections of genetically chloroquine-resistant and -sensitive parasites. Direct sequencing of the *pfcrt* resistance locus and single-nucleotide polymorphism barcoding did not definitively identify a resistant population, suggesting that sustained propagation of chloroquine-resistant parasites was not occurring in Haiti during the study period. Comparison of parasites from Haiti with those from Colombia, Panama, and Venezuela reveals a geographically distinct population with highly related parasites. Our findings indicate low genetic diversity in the parasite population and low levels of chloroquine resistance in Haiti, raising the possibility that reported cases may be of exogenous origin.

## Abstract 2

Low genetic diversity and low levels of chloroquine resistance among parasites suggest exogenous origin of reported cases.

Several decades since malaria has been eradicated from most Caribbean islands, the vectorborne parasitic disease continues to cause sporadic outbreaks in the region and remains endemic only to the island of Hispaniola (*1*), which is the location of the Dominican Republic and Haiti. Evidence suggests that, as in Central America north of Panama, the circulating *Plasmodium falciparum* malaria parasite, which is the dominant malarial species in Haiti and causes illness associated with the highest number of deaths worldwide (http://www.who.int/mediacentre/factsheets/fs094/en/), has remained chloroquine sensitive. The presence of chloroquine-resistant (CQR) parasites in Haiti could have a notable effect on the populace, as well as complicate ongoing efforts of disease control and the ultimate goal of disease eradication. In addition to social and ethical considerations, the eradication of malaria could ultimately aid in alleviating poverty, a particularly critical issue in Haiti, which has the lowest per capita income in the Western Hemisphere (*2*). The known negative effects of malaria on economic growth and human capital development would be amplified because of cost increases for malaria treatment if drug-resistant parasites were to become endemic.

Considering the potential ramifications of the establishment of endemicity of drug-resistant malaria parasites, several surveys have assessed the presence of CQR parasites in Haiti. These studies have predominantly relied upon detection of mutations in the *P. falciparum* chloroquine resistance transporter (*pfcrt*) gene (*3*–*8*) as a proxy for possible drug resistance. Although the presence of a single point mutation does not prove clinical resistance, the substitution at position 76 from lysine (K76) to threonine (T76) is a useful surrogate marker. This technique uses the nested PCR amplification of *pfcrt* gene sequences, which may be easily detected by using DNA extracted from whole blood spotted on filter paper. The filter papers are dried, then stored with desiccant at collection points until conditions for transportation are favorable. Conventionally, the mutation has been detected by mutation-specific restriction–endonuclease digestion of the *pfcrt*-nested PCR fragments with *Apo*I; the gene sequence containing the T76 threonine substitution associated with chloroquine resistance is resistant to digestion, and that containing the wild-type lysine (K76) associated with drug sensitivity reveals 99-bp and 46-bp fragments on agarose gel electrophoresis (*3*–*8*). Because DNA sequencing has become more widely available and routinely practical, direct sequencing of the nested *pfcrt* PCR product for the presence of the mutation has been used (*5*–*8*).

Surveys in Haiti have intermittently detected parasites harboring the CQR haplotype of the *pfcrt* gene (*6*,*9*). In addition, the presence of drug-resistant parasites as assessed by using in vitro culture has been reported (*9*). Clinical chloroquine treatment failures have not been reported, however, and sustained transmission of drug-resistant parasites is not believed to have occurred.

To clarify the genetic context of malaria parasites from Haiti, we used molecular barcode approaches to assess the genetic diversity of this parasite population in the context of geographically distinct neighboring parasite populations. The molecular barcode was used to assess the multiplicity of infection and parasite relatedness. This approach has been used to assess relative parasite transmission intensity (*10**,**11*) and has the potential to track parasites to better understand their relatedness and sources, including in outbreak investigations (*12*). In addition, we genetically characterized a subset of the parasite population in Haiti sampled by single-nucleotide polymorphism (SNP) molecular barcoding to determine their relatedness to other parasite populations in the region.

We report the findings of our surveillance for parasites harboring *pfcrt* CQR haplotypes in patients with suspected malaria at 9 medical sites across Haiti during the 4 years preceding a major earthquake in January 2010. The earthquake destroyed health infrastructure in the country, including the Ministry of Health and Population, killed >220,000 people, and left >1.5 million homeless. In the aftermath of this catastrophe, major efforts were deployed to establish enhanced surveillance systems to detect and prevent the transmission of disease in the affected population (*13**,**14*). 

## Materials and Methods

### Sample Collection and DNA Processing

Surveillance for CQR *P. falciparum* in Haiti was continuous during 2006–2009 by the Haitian Group for the Study of Kaposi’s Sarcoma and Opportunistic Infections (GHESKIO) at 9 healthcare centers in the municipalities of Jeremie, Jacmel, Les Cayes, Miragoane, Cap-Haitien, Deschappelles, Port-au-Prince, Fort-Liberte, and Port-de-Paix. Filter paper (Whatman 3MM, Whatman Corporation, Florham Park, NJ, USA) was cut into 2 cm × 1.5 cm strips. We cut 4 teeth, each with a width of ≈5 mm, in the lower half of the filter paper. Whole blood was obtained by finger prick for absorption on the filter paper teeth (≈50 μL per tooth), and smears from patients with fever and suspected malaria were prepared for parasite detection. The filter papers were dried and stored at room temperature in sealed bags with desiccant. We periodically transferred samples and blood smears to GHESKIO in Port-au-Prince. All blood smears were reviewed at GHESKIO for the presence of parasites. We extracted DNA from the individual filter paper teeth of samples from patients who had parasite-positive smears either by methanol extraction or by using the QIAamp DNA Blood Mini kit (QIAGEN, Valencia, CA, USA) according to the manufacturer’s instructions.

### Molecular Analysis of *pfcrt* Gene Sequences

Initially, we analyzed samples for chloroquine resistance mutations by nested PCR amplification of the *pfcrt* gene, then mutation-specific restriction–endonuclease digestion with *Apo*I as previously described (*3*). Positive and negative controls were included in each round of PCR testing: CQR line Dd2, CQS line 3D7, and water alone. Products were resolved and visualized on a 2% agarose gel. Subsequently, we changed the screening method to the direct sequencing of the nested PCR products, either at the Genomics Core of the Cornell University Life Sciences Core Laboratories Core Facility (http://www.biotech.cornell.edu/node/556) or at Macrogen (Rockville, MD, USA). We cloned PCR products using the TOPO TA Cloning Kit (Life Technologies, Invitrogen, Carlsbad, CA, USA) according to the manufacturer’s instructions.

### Molecular Barcoding

After extraction, molecular barcode data were obtained for a subset of the samples as described previously (*10*). We applied this approach to 72 samples that had sufficient remaining material for processing and included the 2 samples that initially were identified as possibly polygenomic. In brief, 0.3 ng of extracted template material were used in 5 μL total reaction volumes containing TaqMan Universal PCR Master Mix (2×), no AmpErase UNG (Applied Biosystems, Foster City, CA, USA), and 40× TaqMan MGB assays (Applied Biosystems) that were run on a 7900HT real-time system (Applied Biosystems). The samples were genotyped after PCR amplification based on their end-point fluorescence signals (FAM or VIC). Samples that showed >1 mixed-base SNP call or had >5 missing calls in the 24-SNP molecular barcode were removed from analysis.

### Data Analysis

We sorted samples for the presence of identical barcodes. Molecular barcodes that shared >96% of their positions were defined as highly genetically related. For comparison to other populations in the general geographic region, we performed spatial Principal Component Analysis (sPCA) using the adegenet function within the PopGenReport package version 2.0 in R version 3.02 (*15*) to analyze monogenomic parasite barcodes from Panama (n = 37) (*12*), Colombia (n = 7) (*12*), and Venezuela (n = 31) (V. Udhayakumar, pers. comm.). These samples were collected during outbreaks in those countries during 2003–2008, 2011–2012, and 2003–2004, respectively.

## Results

**Figure 1:**
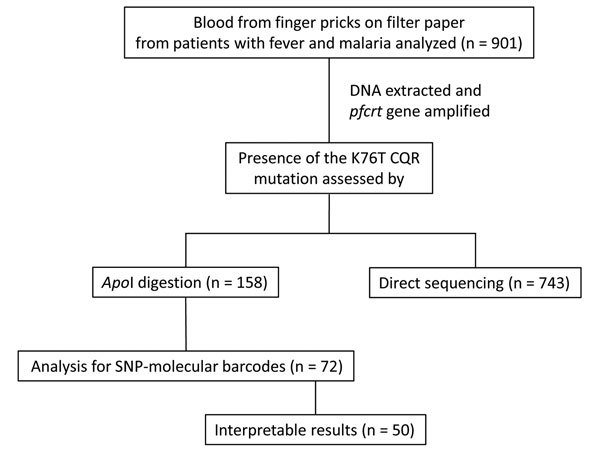
Flowchart of blood specimen processing and analysis for study of presence of *Plasmodium* *falciparum* K76T *pfcrt* gene mutations, Haiti. Each sample was analyzed for the presence of *pfcrt* mutations associated with chloroquine drug resistance by only one method (restriction digest or sequencing) with the exception of 2 samples. *pfcrt*, *P*. *falciparum* chloroquine resistance transporter; CQR, chloroquine-resistant; SNP, single-nucleotide polymorphism.

We analyzed 901 blood samples for the presence of *pfcrt* mutations associated with chloroquine drug resistance (Table 1; Figure 1). Of those, 899 samples were analyzed either by restriction digest or sequencing. Of 158 samples analyzed by *pfcrt* PCR product mutation-specific restriction–endonuclease digestion, 156 showed 99-bp and 46-bp fragments characteristic of the wild-type (chloroquine sensitive) *pfcrt* gene; however, 2 samples repeatedly revealed a fragment resistant to *Apo*I digestion. These samples, 1 each from the cities of Jeremie and Les Cayes, were further analyzed for the possible presence of both chloroquine-sensitive and -resistant parasites. Direct sequencing of the PCR products revealed the potential presence of both alleles. We attempted subcloning of PCR products for these potentially mixed samples to isolate individual PCR products for sequencing, but were not successful. SNP-based molecular barcoding (*10*) revealed the strain from Jeremie to be a unique isolate (Table 2), but results of the analysis for the isolate from Les Cayes could not be interpreted on 2 occasions because of poor amplification. No CQR *pfcrt* mutations were detected among the remaining 743 samples analyzed solely by direct sequencing. The observation that essentially all samples were confirmed to lack the CQR haplotype for *pfcrt* by molecular approaches supports the clinical observation that chloroquine remains highly effective in Haiti.

**Table 1:** Evaluation of *Plasmodium falciparum* samples for chloroquine resistance by restriction-endonuclease digestion and sequencing, Haiti, 2006–2009*

Source	No. samples	No. *pfcrt* K76T *Apo*1 sensitive	No. *pfcrt* K76T *Apo*1 combined	No. *pfcrt* K76T sequenced CVMNK	No. *pfcrt* K76T sequenced CVMNK/CVIET
Jeremie	308	73	1†	234	1†
Jacmel	36	14	0	22	0
Les Cayes	316	40	1‡	275	1‡
Miragoane	15	7	0	8	0
Cap-Haitien	4	2	0	2	0
Deschappelles	199	20	0	179	0
Fort-Liberte	13	ND	NA	13	0
Port-de-Paix	3	ND	NA	3	0
Port-au-Prince	7	ND	NA	7	0
Total	901	156	2	743	2

*Sequenced includes sequencing of the nested PCR product for *pfcrt* haplotypes where wild-type haplotype = CVMNK, and mutant haplotype = CVIET;  ND, not determined; NA, not applicable; *pfcrt*, *P. falciparum* chloroquine resistance transporter. †Same sample from Jeremie. ‡Same sample from Les Cayes; pfcrt K76T *Apo*I includes endonuclease digestion and gel electrophoresis. Sensitive: K76; resistant: 76T; *pfcrt* K76T.

**Table 2:** Monogenic barcoding data of *Plasmodium falciparum* samples determined to assess genetic relatedness, Haiti, 2006 and 2007

Sample	Location	Year	Molecular barcode*	Type	Category
15	Deschappelles	2006	TACTGCGGATTACCACCAAACTTG	A	Identical
35	Les Cayes	2007	TACTGCGGATTACCACCAAACTTG		Identical
22	Jeremie	2007	CACTCCGGATCXTCAACAAACTTG	B	Identical
23	Jeremie	2007	CACTCCGGATCXTCAACAAACTTG		Identical
31	Les Cayes	2007	TACTGGGGACTACACCCTAGCTTG	C	Identical
33	Les Cayes	2007	TACTGGGGACTACACCCTAGCTTG		Identical
38	Les Cayes	2007	TACTCCGGATCGTCCACAAGCTTG	D	Identical
44	Les Cayes	2007	TACTCCGGATCGTCCACAAGCTTG		Identical
27	Les Cayes	2007	TACTCCGGATTXTCCACAAGCTTG		Related
6	Miragoane	2006	TACTCCAGACTXCCAACTCGATTG	E	Related
36	Les Cayes	2007	TACTCCAGACTACCAACTCGATTG		Related
11	Deschappelles	2006	TATCCCGGATTACCACCTAACCTG	F	Related
16	Deschappelles	2007	TATCCCGGATTAXCCCCTAACCTG		Related
30	Les Cayes	2007	TACTGCGGATXACACACTAGCCTG	G	Related
43	Les Cayes	2007	TACTGCGGATTXCACACAAGCCTX		Related
5	Miragoane	2006	XACTCCGGACTXCAACCAAACTTG	U	Unique
7	Miragoane	2006	TACTCCAGACTACCAACTCGACGG	U	Unique
8	Deschappelles	2006	TATTGCGGATTXCCCCCTAACTTG	U	Unique
9	Deschappelles	2006	CACTCCGGATTACACACAAACCTG	U	Unique
10	Deschappelles	2006	TATCCCGGATCATACCCTAACTTG	U	Unique
12	Deschappelles	2006	TACTGCAGATXXCACACTAAACTG	U	Unique
13	Deschappelles	2006	TATTGCAGATCGTCCCCTAGATTG	U	Unique
14	Deschappelles	2006	TACTCCAGATTGTCCCCTAGCTTG	U	Unique
17	Deschappelles	2007	TACTGCGGATCATAACCAAGCTTG	U	Unique
18	Jacmel	2006	TAXTCCGGATTGTCACCAAGCTTG	U	Unique
19	Jacmel	2007	TACTGCGGATCATAAACAAACTTG	U	Unique
20†	Jeremie	2007	TACXCXAGATTXTCXXTACACTTG	U	Unique
21	Jeremie	2007	TATTGCGGATTACACCCTAGCCTG	U	Unique
24	Cap-Haitien	2006	TXCXCCGGAXTXCCCXCAAGXTTG	U	Unique
25	Les Cayes	2006	TACTGCAGATCGTACCTTAGCCTG	U	Unique
26	Les Cayes	2006	TACCCCGGACCGCAACCTAAATTG	U	Unique
28	Les Cayes	2007	TATTCCAGATCGTCCCTTAGACTG	U	Unique
29	Les Cayes	2007	TAXTGCGGATTGTCACCTAACTTG	U	Unique
32	Les Cayes	2007	TACCCCGGATTXCACCCAAACTTG	U	Unique
34	Les Cayes	2007	TACTCCGGACCGCACCCTAAATTG	U	Unique
37	Les Cayes	2007	TACTCCGGACTACCCCCTAACTTG	U	Unique
39	Les Cayes	2007	TATCGCAGATTACCAATAAGCCTG	U	Unique
41	Les Cayes	2007	TATTCCGGATXACCCACTAGCTTG	U	Unique
42	Les Cayes	2007	TATTCGGGATTATCCACTAGATTG	U	Unique
45	Les Cayes	2007	TACXGXAGATTXTCCCCACGCTTG	U	Unique
46	Les Cayes	2007	TACTGCGGACCGXCCCCTAACCTG	U	Unique
47	Les Cayes	2007	TACTGCAGACTXCCCCCACGCTTG	U	Unique
*X indicates missing data, where neither the major or minor allele could be detected. †Sample 20 from Jeremie revealed the possible presence of both alleles by mutation-specific restriction–endonuclease digestion and DNA sequencing.					

**Figure 2:**
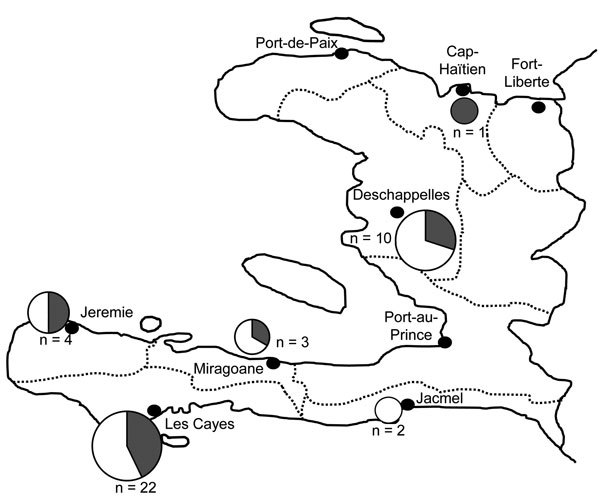
*Plasmodium falciparum* parasite molecular barcode relatedness by site, Haiti. A total of 42 monogenomic samples were obtained from 6 sites (black dots). Circle sizes represent the number of samples from each site. Highly related samples, with either 24/24 or 23/24 identical single-nucleotide polymorphism positions (>96% barcode identity) are shown in gray circle sections; less related samples (<96% barcode identity) are shown in white circle sections.

**Figure 3:**
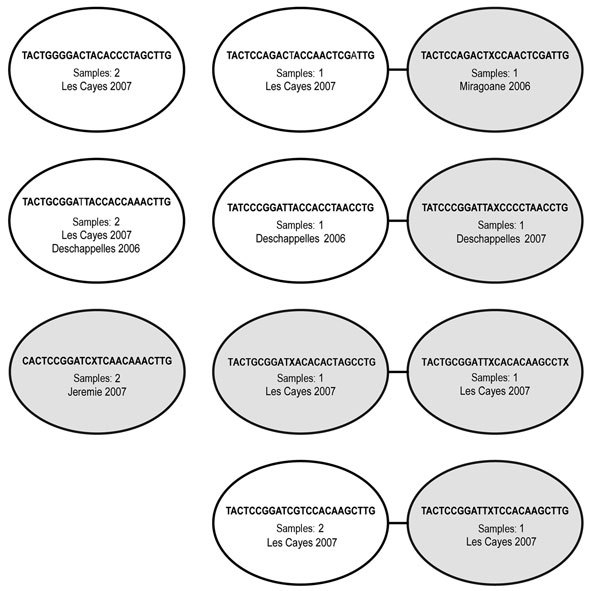
Visualization of 15 identical (same single-nucleotide polymorphism positions call at 24 of 24 positions) and nearly identical (23 of 24 identical positions) molecular barcodes from 42 monogenomic samples from patients in Haiti, 2006 and 2007. Each node (oval) represents an individual barcode. Samples with identical barcodes are included in the same nodes, and related barcodes (1 single-nucleotide polymorphism positions difference) are connected by lines. Gray nodes indicate that there is some point of ambiguity between barcodes, defined as either both alleles detected (N) or no data (X) at a specific position, indicating <100% confidence of a complete match between barcodes.

**Figure 4:**
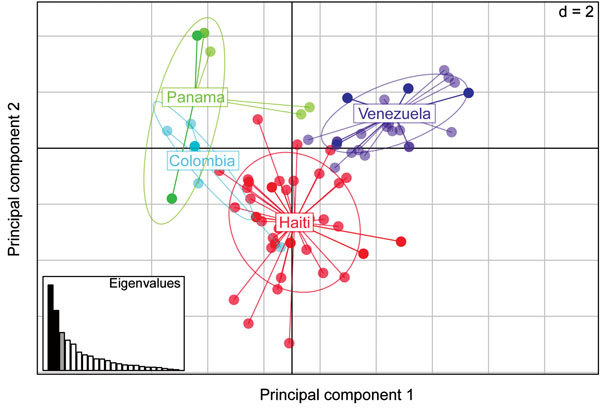
Spatial principal components analysis (sPCA), performed in 2 dimensions (d = 2) comparing malarial parasite population structures based on monogenomic single-nucleotide polymorphism barcodes from Haiti (n = 42), Colombia (n = 7), Panama (n = 37), and Venezuela (n = 31). The x-axis represents the eigenvector associated with the first principal component, which differentiates between populations; the y axis represents the second principal component, which differentiates between samples within the same populations. Inset graph depicts the amount of variability described by the principal components: x-axis indicates individual principal components, y-axis their individual contribution to the observed variance. Black bars, displayed eigenvectors; gray bars, retained principal components; white bars, nonretained principal components.

To better understand the genetic relatedness of these parasites endemic to Haiti and discover whether they are related to or different from neighboring parasite populations, we genotyped a subset of samples with sufficient remaining material using an SNP-based molecular barcoding approach. We used this genotyping method on 72 samples collected in 2006 and 2007; 50 (69%) of these yielded interpretable barcodes; 42 (84%) harbored monogenomic and 8 (16%) polygenomic infections (Tables 2, 3). Further comparative analysis of the 42 monogenomic barcodes revealed a high degree of similarity between the parasite genomes (Figures 2,3). Of the 42 parasites, 15 (36%) had identical or nearly identical molecular barcodes, sharing 23 of 24 positions (96% barcode identity) (Figure 3). Furthermore, some of these nearly genetically identical parasites persisted across transmission seasons from 2006 to 2007 (Figure 3; Table 2). Identical parasite barcodes were identified in Deschappelles in 2006 and Les Cayes in 2007; highly related parasites were also identified in Miragoane in 2006 and Les Cayes in 2007 and in Deschappelles in 2006 and 2007. Additional comparative analysis of the barcodes from Haiti with those from Panama, Colombia, and Venezuela by using sPCA suggest that Haitian parasites are distinct from these other parasite populations in the region (Figure 4). 

**Table 3:** Polygenomic barcodes of *Plasmodium falciparum* samples determined to assess genetic relatedness, Haiti, 2006–2007

Sample	Location	Year	Molecular barcode*
1	Cap-Haitien	2006	TACTGCGGATTNNCCCNAAGCTTG
2	Deschappelles	2006	TATCCCGGATCANACCCTANCNTG
3	Deschappelles	2007	TATCCCNGATCATACCCTAACCTG
4	Deschappelles	2007	TACTNCNGATTNNNCNCAANCNTG
5	Jeremie	2007	TACTNCNGATCGTNACNTANNTTG
6	Les Cayes	2006	CACXGXGXATCXTAAXCTAGNCTG
7	Les Cayes	2007	TATXCXGGATNXCCCCCACGCNTG
8	Les Cayes	2007	TACTCCGGATCGTCCANAAGCTTG
*X indicates missing data, where neither the major or minor allele could be detected; N indicates both alleles detected.			

Although these samples represent a small subset of the malarial parasite population of Haiti, these genetic analyses show highly related parasites within Haiti that are distinct from geographically neighboring parasite populations. Furthermore, detection of parasites with identical barcode genotypes in 2006 and 2007 suggests that parasite populations may persist from one transmission season to another, which implies the need for intervention strategies such as targeting parasite reservoirs that may harbor such persisting parasites from one transmission season to the next.

## Discussion

Malaria remains a major cause of illness and death worldwide. With the exception of Hispaniola, the Caribbean islands are free of sustained *P. falciparum* parasite transmission and report only occasional outbreaks. These outbreaks are likely caused by the importation of parasites by infected persons, leading to local transmission by competent mosquito vectors. However, malaria remains endemic to Haiti and the Dominican Republic on the island of Hispaniola. Consistent with worldwide efforts, the control and eradication of malaria in Hispaniola is deemed warranted and feasible. However, sporadic reports of CQR parasites raise concerns that these efforts may prove more costly and difficult to accomplish than envisioned. 

We report on the analysis of 901 samples from 9 sites throughout Haiti collected during the 4 years preceding the magnitude 7.0 earthquake that occurred in January of 2010. These surveillance efforts were quickly reestablished in the wake of the disaster in order to monitor and prevent the transmission of malaria among vulnerable groups (*15**,**16*). Despite prior reports of the presence of parasites in Haiti harboring the *pfcrt* drug resistance mutation, we found that most of these parasites contained the chloroquine-sensitive *pfcrt* allele. Only 2 samples from southern Haiti contained parasites that possibly harbored a mixed population of parasites, including those containing the allele indicative of drug resistance. An extensive evaluation of these samples from patients in Les Cayes and Jeremie, in which both sensitive and resistant alleles were detected by mutation-specific restriction–endonuclease digestion of nested PCR *pfcrt* gene products, was inconclusive. Direct sequencing of the nested *pfcrt* PCR products yielded variable results but also suggested the presence of resistant and sensitive alleles. Cloning to permit sequencing of unique isolates was unsuccessful, as was molecular barcoding of the sample from Les Cayes. Molecular barcoding of the sample from Jeremie, however, revealed a single strain. These findings might be consistent with the presence of parasite populations subclinically harboring the resistant allele. The presence of such low-density infections may be difficult to detect and characterize; therefore, we cannot conclusively rule out the presence of resistant alleles in these 2 samples (*16*). Nevertheless, considering that only 2 of 901 samples could not be confirmed as harboring only a chloroquine-sensitive haplotype for *pfcrt*, our findings indicate that nearly all parasites tested were chloroquine sensitive. We therefore conclude that chloroquine resistance was not likely to be present in Haiti during the analyzed period and that chloroquine remains clinically useful in Haiti.

The observation that increasing use of chloroquine after the earthquake in 2010 did not select for or increase the prevalence of these mutations among the parasite population as described by Morton et al. (*14*) is consistent with the lack of resistant forms of *pfcrt* among the population sampled in 2006 and 2009 in Haiti. Although a 2011–2012 study conducted by Okech et al. raises concerns regarding drug efficacy related to the persistence of parasites in some patients treated with chloroquine, the resistance mutation was not detected (*17*). A prior report indicated a high percentage of *pfcrt* mutant alleles (5/79, 6%) in *P. falciparum*–positive blood samples in the Artibonite Valley of Haiti in 2006 and 2007 (*6*). Our surveillance included 199 samples from this region but did not show resistant alleles, indicating the transmission in this region may not have been sustained under chloroquine pressure. However, possible resistance cannot be definitively excluded.

We used the molecular barcode data for parasite population genetic analysis; results suggest that the *P. falciparum* parasite population in Haiti is highly related genetically. Of the 42 samples analyzed, 15 (36%) shared >23 of the 24 SNPs of the molecular barcode (96% identical barcode). We have shown in a previous study comparing relationships of the molecular barcode and whole-genome sequencing data that molecular barcodes that share >75% of their positions are highly genetically related (*11*), indicating that samples described here as identical at 23 or 24 markers in the molecular barcode are genetically or nearly genetically identical.

We increased the threshold to 96% in this study to increase our confidence of identifying truly related parasite types. Furthermore, specific parasite types persist from one year to the next among these highly related parasites, suggesting transmission of single clones without recombination and likely limited introduction of new parasite types through travel or migration. These observations of highly related and clonal parasites that endure across years are consistent with decreasing transmission and potential inbreeding among the parasite population in Haiti. They are also consistent with the population structure analysis of Morton et al. (*14*). 

The PCA analysis reveals that the *P. falciparum* parasite population in Haiti is generally separate from and independent of parasite populations from Panama, Colombia, and Venezuela. However, 1 parasite from Colombia clusters with the parasite population in Haiti. This observation is consistent with the possible transfer of parasites between this site and Haiti. However, these observations are based on a relatively limited number of samples; although we cannot rule out potential sampling bias, these findings are noteworthy.

After a temporary lull in control efforts related to *P*. *falciparium* malaria after the 2010 earthquake, attempts at control with an ultimate goal of eradication have resumed (*18*,*19*). Characterization of baseline populations before catastrophic events can support efforts to identify and predict the effects on parasite populations in response to relaxation of control efforts and changed environments. In Haiti, identification of specific parasite genetic types may suggest importation of parasites related to humanitarian efforts or population migration; in addition, the identification of hot spots of transmission of specific parasite types can direct precise application of control efforts, particularly when resources are limited. Our equivocal detection of only 2 patients potentially harboring low-level populations of chloroquine resistance alleles despite screening >900 samples collected during 4 years supports the contention that the sustained propagation of CQR parasites was not occurring in Haiti during the study period; the possibility is raised by Morton et al. that reported cases are of exogenous origin (*14*). The finding of low genetic diversity in the *P. falciparum* population in Haiti is also consistent with that of Morton et al. and others (*7*,*14*,*20*). Although encouraging, these findings support the need for continued surveillance during eradication efforts as well as additional studies to better understand the malarial parasite population structure in Haiti.
